# Prognostic Relevance of the Proximal Resection Margin Distance in Distal Gastrectomy for Gastric Adenocarcinoma

**DOI:** 10.1245/s10434-024-15721-y

**Published:** 2024-07-05

**Authors:** Ingmar F. Rompen, Isabel Schütte, Nerma Crnovrsanin, Sabine Schiefer, Adrian T. Billeter, Georg Martin Haag, Thomas Longerich, Zoltan Czigany, Thomas Schmidt, Franck Billmann, Leila Sisic, Henrik Nienhüser

**Affiliations:** 1 Department of General, Visceral and Transplantat Surgery, Heidelberg University Hospital, Heidelberg, Germany; 2 Department of Surgery, Clarunis-University Digestive Health Care Center, St. Clara Hospital and University Hospital Basel, Basel, Switzerland; 3 Department of Medical Oncology, National Center for Tumor Diseases (NCT), Heidelberg University Hospital, Heidelberg, Germany; 4 Institute of Pathology Heidelberg, Heidelberg University Hospital, Heidelberg, Germany; 5 Department of General, Visceral, Cancer and Transplant Surgery, University Hospital of Cologne, Cologne, Germany

**Keywords:** Gastric neoplasm, Gastric cancer, Resection margin, Margin distance, Surgery, Distal gastrectomy

## Abstract

**Background:**

The risk for recurrence in patients with distal gastric cancer can be reduced by surgical radicality. However, dispute exists about the value of the proposed minimum proximal margin distance (PMD). Here, we assess the prognostic value of the safety distance between the proximal resection margin and the tumor.

**Patients and Methods:**

This is a single-center cohort study of patients undergoing distal gastrectomy for gastric adenocarcinoma (2001–2021). Cohorts were defined by adequacy of the PMD according to the European Society for Medical Oncology (ESMO) guidelines (≥ 5 cm for intestinal and ≥ 8 cm for diffuse Laurén’s subtypes). Overall survival (OS) and time to progression (TTP) were assessed by log-rank and multivariable Cox-regression analyses.

**Results:**

Of 176 patients, 70 (39.8%) had a sufficient PMD. An adequate PMD was associated with cancer of the intestinal subtype (67% vs. 45%, *p* = 0.010). Estimated 5-year survival was 63% [95% confidence interval (CI) 51–78] and 62% (95% CI 53–73) for adequate and inadequate PMD, respectively. Overall, an adequate PMD was not prognostic for OS (HR 0.81, 95% CI 0.48–1.38) in the multivariable analysis. However, in patients with diffuse subtype, an adequate PMD was associated with improved oncological outcomes (median OS not reached versus 131 months, *p* = 0.038, median TTP not reached versus 88.0 months, *p* = 0.003).

**Conclusion:**

Patients with diffuse gastric cancer are at greater risk to undergo resection with an inadequate PMD, which in those patients is associated with worse oncological outcomes. For the intestinal subtype, there was no prognostic association with PMD, indicating that a distal gastrectomy with partial preservation of the gastric function may also be feasible in the setting where an extensive PMD is not achievable.

**Supplementary Information:**

The online version contains supplementary material available at 10.1245/s10434-024-15721-y.

Gastric cancer is the fifth most common cancer worldwide with significant regional differences in incidence and mortality.^1^ In Western countries with lower incidence than in Asia, screening is not routinely performed, leading to relatively more advanced tumor stages at time of diagnosis.^2^ In those patients with tumors infiltrating the submucosa or beyond, surgical resection with D2 lymphadenectomy is recommended.^3–5^ For distal gastric cancer, surgical resection can be performed by either distal gastrectomy or total gastrectomy.^6^ Preserving the proximal stomach is associated with lower risk for postoperative complications and portends better functional outcome as well as improved quality of life as compared with total gastrectomy.^7,8^

However, even after surgery in curative intent, approximately one-third of patients experience recurrence at a local or, predominantly, at a distant site.^9,10^ This is driven by locally advanced tumor stages and a high risk of lymphatic and hematogenous dissemination.^10–12^ Consequently, in addition to providing systemic treatment, the risk for recurrence can be modified by the radicality of surgical treatment with an adequate lymphadenectomy and negative resection margins.^10,13–15^ Dispute, however, exists about the optimal distance of the surgical margins to the tumor to both ensure an adequate removal of the tumor itself and facilitate the removal of the regional lymph nodes draining the tumor.^13,16^ This dispute has led to differing guidelines regarding the feasibility of distal gastrectomy as an acceptable treatment option for gastric cancer within the distal stomach.^17^ The European Society for Medical Oncology (ESMO) guidelines state that a distal gastrectomy is feasible if a macroscopic proximal margin distance (PMD) of 5 cm can be achieved for intestinal type cancers.^5^ For diffuse cancers, even a wider PMD of 8 cm is advocated, with otherwise a total gastrectomy being indicated.^5^ Asian guidelines such as the Japanese or Chinese gastric cancer associations (JGCA, CSCO), however, recommend obtaining at least a 3 or 5 cm PMD depending on its gross appearance (Borrmann types).^3,4^

This study aims to assess the prognostic value of the safety distance between the proximal resection margin and the tumor.

## Patients and Methods

This analysis was performed by a single-center cohort study of patients undergoing distal gastrectomy for gastric adenocarcinoma. Patient data were extracted from a prospectively maintained database. Ethical approval was obtained by the Ethics Committee of Heidelberg University (S-649-2012). The study was performed in compliance with the Strengthening the Reporting of Observational Studies in Epidemiology (STROBE) guidelines and complies with the 1964 Helsinki Declaration and its later amendments.^18^ All included patients filed informed consent for data use upon treatment.

Patients who underwent distal gastrectomy for gastric adenocarcinoma at the Heidelberg University Hospital between 2001 and 2021 were included. Exclusion criteria encompassed tumor entity other than intestinal, mixed, or diffuse adenocarcinoma, macroscopically positive resection margins (R2), patients undergoing total gastrectomy or multivisceral resections, and patients with insufficient follow-up (no follow-up after discharge) or missing data on primary outcomes.

The decision on total versus partial (distal) gastrectomy was made independently of this study by radiologic imaging, endoscopic, and intraoperative assessment. All patients received a gastroscopy prior to resection to determine the localization of the tumor and the histologic subtype. Reconstruction after distal gastrectomy was performed with an end-side gastrojejunostomy and Roux-en-Y reconstruction or, alternatively, according to Billroth II. A frozen section of the proximal margin was performed intraoperatively, and re-resection or completion gastrectomy was performed if positive. The latter patients were excluded from final analysis. A D2-lymphadenectomy was standard of care in all patients resected for gastric adenocarcinoma. Patients were followed up with regular clinical visits and serologic, radiographic and endoscopic diagnostics until 60 months postoperatively.^19^ Recommended follow-up included assessment of recurrence by CT imaging and endoscopy in alternation every 3 months within the first 2 postoperative years and every 6 months thereafter.^20,21^ Further follow-up data were obtained through electrical records of clinical visits, telephone interviews with patients or their primary care provider, and death certificates.

Overall survival (OS) and time to progression (TTP) were defined as primary outcome parameters. Survival was defined as the time from diagnosis to death (OS) or recurrence (TTP). When the time of diagnosis was not available, the date of first treatment was used. The AJCC/UICC 8th edition was used for TNM staging.^22^ Treatment response was assessed according to the Becker classification.^23^ Analyses were performed separately for tumor types classified by the Laurén’s and updated in the European Chapter of International Gastric Cancer Association.^24,25^ Therefore, cohorts were formed according to the presence of < 10%, 10–90%, and ≥ 90% signet ring cells for intestinal type, mixed, and diffuse/poorly cohesive type (further referred to as diffuse within this manuscript), respectively.^24^ Patients with mixed and diffuse subtypes were grouped for final analysis. A PMD was deemed adequate when exceeding 5 cm for intestinal or 8 cm for mixed/diffuse type cancers. Subanalyses were performed on different cutoffs as proposed by the JGCA. The optimal PMD was assessed by the most significant difference in OS using log-rank estimates (similar to the lowest *p*-value method). Assessment of the PMD was performed by measuring the macroscopic tumor margin to the nearest point of the proximal resection margin in formalin-fixed pathological specimens.

Differences in the distribution of categorical data were compared using the chi-square test, and continuous data were compared using the Student’s *t*-test. Data are presented with median and interquartile range (IQR) or mean and standard deviation (SD) as deemed appropriate. The Kruskal–Wallis test was used for comparisons of multiple groups with nonparametric data. Missing data were mentioned in the tables and removed for group comparisons. Uni- and multivariable Cox-regression analysis was used to identify variables associated with OS and TTP. Variables with prognostic relevance (*p* < 0.10) were assessed in the multivariable analysis. Hazard ratios (HR) and 95% confidence intervals (95% CI) were calculated for each variable. Survival comparisons were visualized using the Kaplan–Meier curves. Subgroup analyses were performed for nonmetastatic patients that were nodal negative or nodal positive and patients after R0 resections. A two-sided *p*-value of < 0.05 was considered statistically significant. Statistical analysis was performed using R (version 4.2) using the Surfminer, Gtsummary, and Ggplot2 packages.

## Results

### Study Cohort

A total of 806 patients were treated for distal gastric adenocarcinoma at the Surgical Department of the Heidelberg University Hospital. After serial exclusion of patients without resection after exploration (*n* = 61), patients with gross macroscopic resection margins (R2, *n* = 4), total gastrectomy (*n* = 418), multivisceral resection (*n* = 18), missing data on PMD (*n* = 107), or insufficient follow-up (*n* = 19), 179 patients met the inclusion criteria. Of those, another three patients were excluded due to having a positive frozen section and consecutively underwent completion gastrectomy.

Therefore, a total of 176 patients were included for final analysis. Median follow-up for surviving patients was 68.5 months. Ninety-five patients were diagnosed with intestinal type cancer, 29 had mixed type, and 52 diffuse gastric adenocarcinomas (Supplementary Table 1). Major morbidity (Clavien–Dindo ≥ 3) was observed in 9.7% of patients after distal gastrectomy, with 3.4% showing leakage of the gastro-jejunal anastomosis. Histopathological assessment revealed 15% microscopically positive resection margins, only one (0.6%) at the proximal gastric margin. The mean PMD was 5.2 cm (SD 2.9). Patterns of recurrence were 15% local, 4% peritoneal, and 47% systemic as first site of recurrence, while 35% had multiple of the aforementioned sites involved. No significant differences were observed when comparing the subtypes (*p* = 0.467, Supplementary Fig. 1).

### Proximal Resection Margin Distance in the Overall Cohort

**Fig. 1:**
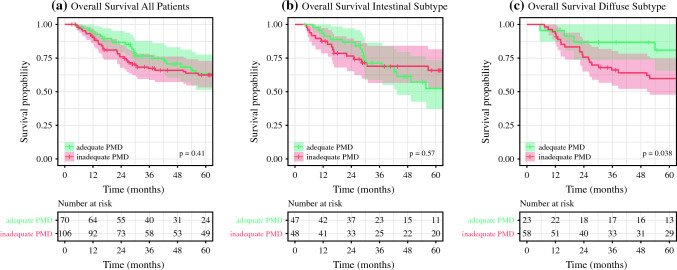
Overall survival by proximal margin distance. Overall survival stratified by adequate and inadequate proximal resection margin distance (PMD) according to the ESMO guidelines [5 cm for intestinal subtype (**b**) and 8 cm for mixed and diffuse subtype (**c**) according to the Laurén classification]. Unstratified analysis only shows a survival difference for patients with diffuse or mixed subtype

**Fig. 2:**
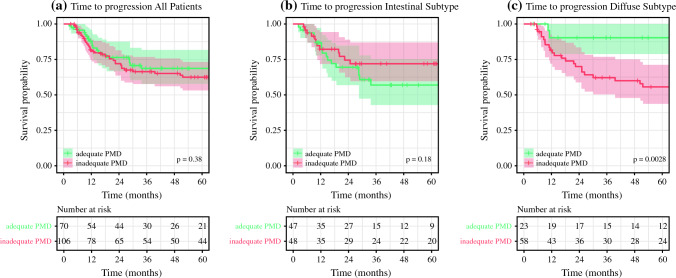
Time to recurrence by proximal resection margin distance. Time to progression (TTP) stratified by adequate and inadequate proximal resection margin distance (PMD) according to the ESMO guidelines [5 cm for intestinal subtype (**b**) and 8 cm for mixed and diffuse subtype (**c**) according to the Laurén classification]. Similarly to OS, unstratified analysis only shows a survival difference for patients with diffuse or mixed subtype

**Fig. 3:**
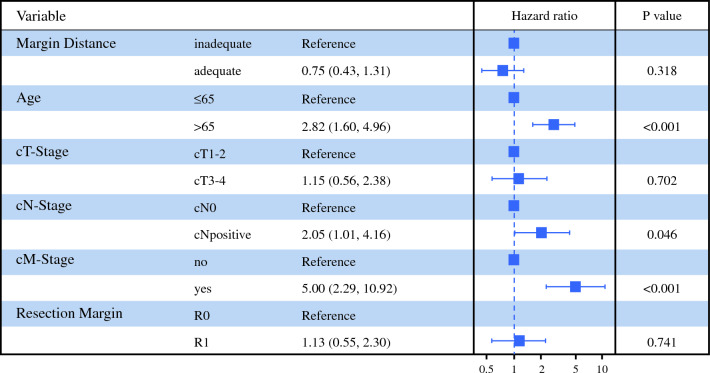
Multivariable Cox regression analysis overall survival. Multivariable Cox regression analysis shows that, after adjusting for age, and pathological TNM stages, the proximal resection margin distance is not an independent prognostic factor

According to the ESMO guidelines (5 cm for intestinal, 8 cm for diffuse), 70 patients (39.8%) had a sufficient PMD. An adequate distance was associated with negative resection margins (93% vs. 79%, *p* = 0.014) and intestinal subtype (67% vs. 45%, *p* = 0.010). There were no differences in other clinicopathological characteristics, notably not in receipt of neoadjuvant treatment (34% vs. 40%, *p* = 0.474), cT category (*p* = 0.609), or mean number of resected lymph nodes (27.8 vs. 27.3, *p* = 0.320, Table 1) between patients with an adequate versus an inadequate PMD. Also, no difference in OS (adequate PMD; median not reached versus inadequate PMD; 131 months, *p* = 0.406, Fig. 1a) and TTP (median not reached versus 159 months, *p* = 0.375, Fig. 2a) was observed. Estimated 5-year survival was 63% (95% CI 51–78) and 62% (95% CI 53–73) for adequate and inadequate distance, respectively. Recurrence was observed in 29% of patients with adequate PMD and 37% of patients with inadequate PMD (*p* = 0.258). In the cohort with adequate PMD, 26% of recurrences were local, 42% systemic, and 11% peritoneal and 21% had multiple sites of recurrence, whereas a different distribution was observed in the cohort with inadequate PMD with 8.3% local, 50% systemic, and 42% multiple (*p* = 0.041). The overall rate of local recurrence (including patients with multiple sites) was 11% and 6.6% (*p* = 0.262) for adequate and inadequate PMD, respectively. After adjusting for age, clinical TNM stage, and resection margin in the multivariate Cox-regression analysis, adequate PMD was not a prognostic factor for OS (HR 0.75, 95% CI 0.43–1.31, Fig. 3) or TTP (HR 0.96, 95% CI 0.53–1.74, Supplementary Fig. 2).

**Table 1:** Resection margin distance according to the ESMO guidelines

Variables	All patients, *N* = 176^a^	Inadequate, *N* = 106^a^	Adequate, *N* = 70^a^	*p*-Value^b^
Age	64.85 (12.60)	63.79 (12.71)	66.44 (12.36)	0.131
Sex (female)	65 (37%)	44 (42%)	21 (30%)	0.122
ASA				0.424
1/2	84 (48%)	48 (45%)	36 (51%)	
3/4	92 (52%)	58 (55%)	34 (49%)	
Subtype				**0.010**
Intestinal	95 (54%)	48 (45%)	47 (67%)	
Mixed	29 (16%)	23 (22%)	6 (8.6%)	
Diffuse	52 (30%)	35 (33%)	17 (24%)	
cT stage				0.609
1/2	70 (42%)	41 (41%)	29 (45%)	
3/4	96 (58%)	60 (59%)	36 (55%)	
Unknow	10	5	5	
cN stage				0.492
Negative	85 (50%)	49 (48%)	36 (53%)	
Positive	85 (50%)	54 (52%)	32 (47%)	
Unknown	5	3	2	
Oligometastatic disease	16 (9.1%)	13 (12%)	3 (4.3%)	0.072
Neoadjuvant treatment	66 (38%)	42 (40%)	24 (34%)	0.474
Major complication	17 (9.7%)	8 (7.5%)	9 (13%)	0.243
Anastomotic leak	6 (3.4%)	3 (2.8%)	3 (4.3%)	0.681
Lymph nodes resected	27.49 (12.53)	27 (14)	28 (11)	0.320
Lymph node ratio	0.15 (0.23)	0.16 (0.23)	0.14 (0.23)	0.107
Resection margin				**0.014**
R0	149 (85%)	84 (79%)	65 (93%)	
R1	27 (15%)	22 (21%)	5 (7.1%)	
Grade				0.113
Well/moderate	58 (35%)	31 (31%)	27 (43%)	
Poor	106 (65%)	70 (69%)	36 (57%)	
Unknown	12	5	7	
Regression				0.147
TRG 1a (pCR)	3 (4.6%)	2 (4.9%)	1 (4.2%)	
TRG 1b	12 (18%)	5 (12%)	7 (29%)	
TRG 2	20 (31%)	16 (39%)	4 (17%)	
TRG 3	30 (46%)	18 (44%)	12 (50%)	
No neoadjuvant or unknown	111	65	46	
Adjuvant treatment	79 (47%)	49 (48%)	30 (45%)	0.743
Unknown	8	4	4	
Recurrence	59 (34%)	39 (37%)	20 (29%)	0.258
Site of recurrence				**0.041**
Local	8 (15%)	3 (8.3%)	5 (26%)	
Multiple	19 (35%)	15 (42%)	4 (21%)	
Peritoneal	2 (4%)	0 (0%)	2 (11%)	
Systemic	26 (47%)	18 (50%)	8 (42%)	
No recurrence or unknown site	121	70	51	

*TRG* Tumor regression grade (Becker classification), *pCR* complete pathological response

^a^*n* (%); mean (SD)

^b^Pearson’s chi-squared test; Wilcoxon rank sum test; Fisher’s exact test for intestinal versus diffuse subtype. Diffuse and mixed subtypes are combined

*p*-values < 0.05 were considered as significant bold values

Similar results were found for patients after neoadjuvant treatment, with 24 out of 66 patients having an adequate PMD. The cohort with an adequate PMD had more intestinal type cancer (83% versus 43%, *p* = 0.005), but no other differences were observed in clinicopathological features compared with the cohort with an inadequate PMD. Again, no significant difference in survival was observed (Median OS (mOS) adequate PMD; not reached versus inadequate PMD; 131 months, *p* = 0.867, mTPP not reached versus 125 months, *p* = 0.954, estimated 5-year survival 62% versus 55%). No survival differences were found for inadequate versus adequate PMD in subgroup analyses for node negative or node positive or after excluding patients with an R1 resection elsewhere (Supplementary Text 1–3).

### Proximal Resection Margin Distance in the Intestinal Subtype

Forty-seven out of 95 patients (48.0%) with intestinal type cancer had a resection margin length of 5 cm and more. There was no significant difference in clinicopathological characteristics between the subgroups according to adequacy of the PMD (Supplementary Table 2). Unadjusted survival analysis revealed no significant difference in median OS (adequate PMD; 94.8 months versus inadequate PMD; median not reached, *p* = 0.572, Fig. 1b) or median TTP (66.3 months versus median not reached, *p* = 0.177, Fig. 2b). Estimated 5-year survival was 52% (95% CI 37–74) and 66% (95% CI 53–82), and recurrence was observed in 38% and 25% of patients (*p* = 0.163) for adequate and inadequate PMD, respectively.

Also, patients with less than 3 cm (JGCA guidelines, *n* = 24) had similar survival compared with those with more than 3 cm (*n* = 71) PMD (mOS not reached versus 94.8 months, *p* = 0.717, median time to progression (mTTP) both not reached, *p* = 0.795, estimated 5-year survival 61% versus 59%). No PMD cutoff was associated with a significant difference in survival for intestinal subtypes (lowest *p*-value > 0.05).

### Proximal Resection Margin Distance in Diffuse and Mixed Subtypes

Seventeen out of 52 patients (32.7%) with diffuse type and 6 out of 29 (20.7%) with mixed type cancer had a PMD of more than 8 cm (ESMO guidelines). Combined, 28.4% had an adequate resection margin. Comparing the cohorts with adequate versus inadequate PMD, there were significant differences in neoadjuvant treatment (17% vs. 41%, *p* = 0.041), clinical T stage (cT3/4; 36% vs. 65%, *p* = 0.020), cN stage (cN+; 27% vs. 55%, *p* = 0.025), mean lymph node ratio (0.07 vs. 0.19, *p* = 0.007), and positive resection margins (R1: 4.3% vs. 31%, *p* = 0.011). All clinicopathological characteristics are presented in Supplementary Table 3. An adequate PMD was associated with better survival (mOS not reached vs. 131 months, *p* = 0.038 and estimated 5-year OS 81% vs. 60%, Fig. 1c) and better disease-specific survival (mTTP not reached vs. 88.0 months, *p* = 0.003, Fig. 2c). Recurrence was observed in 8.7% with adequate PMD and 47% in the inadequate PMD cohort (*p* = 0.001).

A total of 45 patients (55.6%) had more than 5 cm PMD (JCGA guidelines). However, no significant difference in OS (mOS not reached versus 88.0 months, *p* = 0.306) or TTP (mTTP not reached versus 88.0 months, *p* = 0.097) was observed. Estimate 5-year survival was 68% (95% CI 55–84) and 64% (95% CI 49–83) for adequate and inadequate PMD, respectively. The optimal cutoff to show a difference in OS was equal to the ESMO guidelines with 8 cm (lowest *p*-value 0.038).

## Discussion

In this analysis, distal gastrectomy has been shown to be a valuable approach in the treatment of distal gastric cancer with excellent postoperative outcomes and survival. After routine intraoperative frozen section, only one patient had a R1 resection at the proximal resection margin showing adequacy of intraoperative assessment. Still, an adequate PMD according to the ESMO guidelines was associated with fewer R0 resections at other sites. Also, this analysis shows that obtaining an adequate resection margin of 8 cm in diffuse type gastric adenocarcinoma is less common in clinical practice as 5 cm for intestinal type cancers. Overall and for intestinal subtypes, the PMD has no effect on survival outcomes or the rate of local recurrences. In cancers with noncohesive growth patterns (diffuse and mixed type), the PMD of 8 cm was associated with better OS and TTP as well as fewer recurrences.

Distal gastrectomy as a treatment portends excellent oncological outcomes as evidenced by the two out of three patients surviving at least 5 years despite inclusion of patients with locally advanced tumor stages and oligometastatic disease. These results are comparable to other series on distal gastrectomy in the treatment of gastric adenocarcinoma.^26^ Furthermore, distal gastrectomy may be preferred to total gastrectomy to partly preserve gastric function.^8^ However, distal gastrectomy is limited by the feasibility of a complete oncological resection due to possible extensive proximal tumor extension.^27^ For this, surgical radicality for obtaining a negative resection margin and removal of sufficient regional lymph nodes is crucial.^6^ While the distal transection typically is performed at the first part of the duodenum, an elaborative surgical approach is warranted for the proximal transection line in partial gastrectomy in the treatment of distal gastric tumors. Correlations of less R1 resections with a greater safety distance between the tumor and the proximal transection level have led to integration of the safety distance in common guidelines.^28,29^ However, with improvement of intraoperative frozen section guaranteeing a negative proximal margin, several studies have retrospectively questioned the oncological benefit of an extensive PMD. Squires et al. have found no significant survival benefit for more than 5 cm safety distance as compared with 3 cm.^16^ However, a distance of 0–3 cm was associated with worse overall survival.^16^ In multivariable analysis, the effect was shown only for stage I tumors but not for stage II and III gastric cancer.^16^ Similar to the overall effects found in this analysis, two Asian studies found no association of the PMD to oncological outcomes such as overall survival and recurrence.^30,31^ This was also concluded in a study by Lee et al. for gastric cancer in the middle third of the stomach and by Postlewait et al. for proximal gastric cancer with similar survival outcomes and rates of local recurrence.^32,33^

These results would suggest that distal gastrectomies could be a valuable treatment option also if the proximal safety distance cannot be achieved. In this study, this implication can only be made for intestinal type cancers. However, this does not apply for diffuse type cancers in which an inadequate PMD was associated with a decreased disease-specific and overall survival. This effect was also shown in patients with R0 resections at all margins. As shown by Berlth et al., diffuse histology is a risk factor for underestimation of the PMD.^34^ This is also shown with a lower rate of adequate PMD when comparing diffuse with intestinal type cancers in this study. Only one patient had a false-negative frozen section resulting in a positive proximal margin ensuring complete tumor resection at the proximal margin. However, a larger PMD can facilitate lymphadenectomy of perigastric lymph nodes proximal to the tumor. As lymph node mapping studies have shown multidirectional lymph flow and variance in distribution of involved lymph nodes with gastric cancer, a greater distance of the resection margin to the tumor could improve staging and may reduce locoregional lymph node recurrences.^35,36^ Therefore, especially in diffuse type cancers where intraoperative localization may be challenging, better localization through intraoperative gastrofibroscopy or marking clips may be beneficial.^37^

Certain limitations are inherent to the study design and assessment of the PMD. First, this study was based on the assessment of the PMD by the pathologist after formalin fixation. The discrepancy to the stretched intraoperative specimen compared with the shrunk specimen that was measured may result in underestimation of the PMD.^38^ Also, the degree of shrinkage depends on the time of formalin fixation, which cannot be controlled in a retrospective study. Second, while some correlations exist between Borrmann type and the Laurén classification, comparisons between Asian studies based on the former and Western studies based on the latter may be inadequate.^39^ Last, patients with total gastrectomies or intraoperative positive frozen section resulting in secondary total gastrectomy were excluded from this study owing to selection bias that cannot be compensated in a nonrandomized analysis. This may have contributed to a limited sample size hampering conclusion on some subanalyses. In those, neoadjuvant treatment or the presence of nodal disease had a limited effect on the prognostic relevance of the PMD, implicating applicability to patients of all stages.

## Conclusions

Patients with diffuse gastric cancer are at greater risk to undergo resection with an inadequate proximal margin distance. An oncological benefit of an extensive proximal margin distance according to the ESMO guidelines (≥ 8 cm) was found only for patients with diffuse and mixed type gastric cancer. For intestinal type gastric cancer, there was no prognostic association with a proximal margin distance of 5 cm or any other cutoff. These results implicate that in those patients a distal gastrectomy with partial preservation of the gastric function may also be feasible in the setting where an extensive margin distance is not possible. A prospective randomized controlled trial may be necessary to validate these assumptions.

## Supplementary Information

Below is the link to the electronic supplementary material.

Supplementary file1 (DOCX 238 kb)
