# Supplementary material for: Prognostic Relevance of the Proximal Resection Margin Distance in Distal Gastrectomy for Gastric Adenocarcinoma
Source: Ann Surg Oncol. 2024 Jul 5;31(10):6900–8. doi: 10.1245/s10434-024-15721-y (PMC11413044; doi:10.1245/s10434-024-15721-y)
Supplement: Supplementary file 1 — Supplementary file1 (DOCX 238 kb) [file 10434_2024_15721_MOESM1_ESM.docx]

**Supplemental Figure 1: Patterns of Recurrence**

**Supplemental Figure 2: Multivariable Cox-Regression Analysis for Time to Progression**


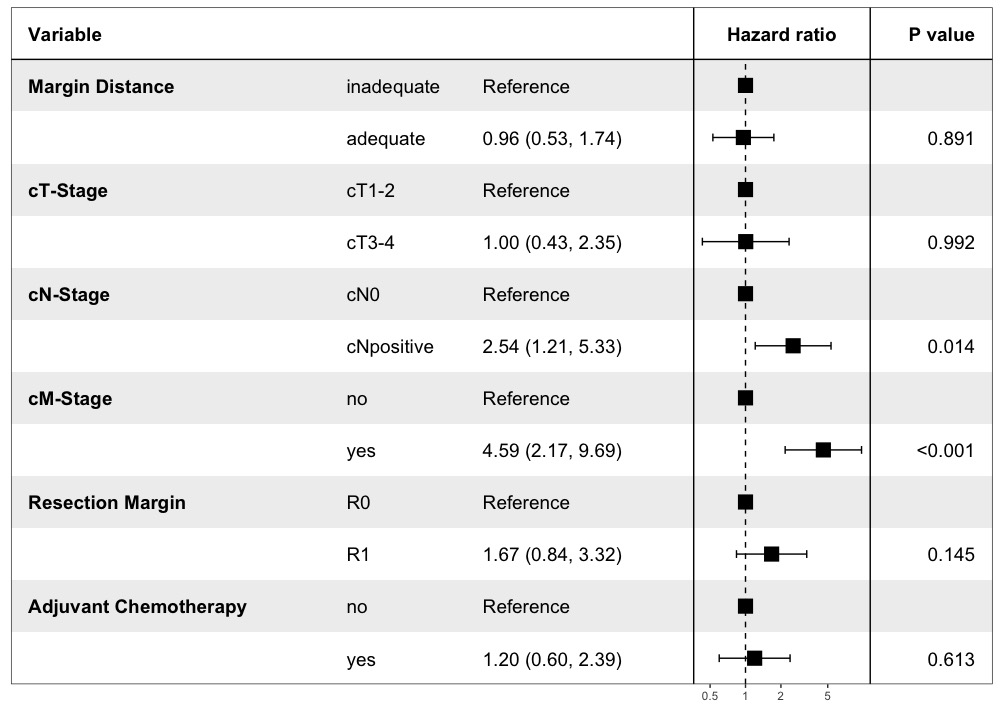


| Supplementary Table 1: Subtype Comparisons | intestinal, N = 95*^1^* | mixed, N = 29*^1^* | diffuse, N = 52*^1^* | p-value*^2^* |
| --- | --- | --- | --- | --- |
| ASA |  |  |  | 0.023 |
| 1/2 | 38 (40%) | 13 (45%) | 33 (63%) |  |
| 3/4 | 57 (60%) | 16 (55%) | 19 (37%) |  |
| Age | 68.80 (11.07) | 63.97 (12.61) | 58.12 (12.49) | <0.001 |
| Sex (female) | 33 (35%) | 12 (41%) | 20 (38%) | 0.781 |
| Grade |  |  |  | <0.001 |
| well-moderate | 52 (61%) | 6 (21%) | 0 (0%) |  |
| poor | 33 (39%) | 22 (79%) | 51 (100%) |  |
| Unknown | 10 | 1 | 1 |  |
| cT-Stage | |  |  | 0.735 |
| cT1 | 11 (12%) | 2 (7.1%) | 7 (14%) |  |
| cT2 | 26 (29%) | 9 (32%) | 15 (31%) |  |
| cT3 | 43 (48%) | 16 (57%) | 20 (41%) |  |
| cT4 | 9 (10%) | 1 (3.6%) | 7 (14%) |  |
| Unknown | 6 | 1 | 3 |  |
| cN-Stage | |  |  | 0.564 |
| cN0 | 44 (47%) | 17 (59%) | 24 (49%) |  |
| cNpositive | 49 (53%) | 12 (41%) | 25 (51%) |  |
| Unknown | 2 | 0 | 3 |  |
| Neoadjuvant Treatment | 38 (40%) | 10 (34%) | 18 (35%) | 0.759 |
| Complication | 8 (8.4%) | 5 (17%) | 4 (7.7%) | 0.318 |
| Anastomotic Leakage | 4 (4.2%) | 1 (3.6%) | 1 (1.9%) | 0.86 |
| Unknown | 0 | 1 | 0 |  |
| Lymph Nodes Resected | 28.88 (13.46) | 25.97 (12.89) | 25.79 (10.29) | 0.36 |
| Proximal Margin Distance | 4.91 (2.83) | 4.98 (2.92) | 5.73 (3.01) | 0.223 |
| pT-Stage | |  |  | 0.191 |
| pT0-1 | 31 (40%) | 5 (22%) | 14 (31%) |  |
| pT2 | 0 (0%) | 0 (0%) | 0 (0%) |  |
| pT3 | 37 (48%) | 15 (65%) | 20 (44%) |  |
| pT4 | 9 (12%) | 3 (13%) | 11 (24%) |  |
| Unknown | 18 | 6 | 7 |  |
| pN-Stage | |  |  | 0.92 |
| pN0 | 44 (46%) | 13 (45%) | 24 (46%) |  |
| pN1 | 13 (14%) | 5 (17%) | 10 (19%) |  |
| pN2 | 17 (18%) | 3 (10%) | 7 (13%) |  |
| pN3 | 21 (22%) | 8 (28%) | 11 (21%) |  |
| pM | 7 (7.4%) | 2 (6.9%) | 7 (13%) | 0.394 |
| Lymph Node Ratio | 0.15 (0.22) | 0.15 (0.23) | 0.16 (0.25) | 0.973 |
| Resection Margin | |  |  | 0.011 |
| R0 | 87 (92%) | 24 (83%) | 38 (73%) |  |
| R1 | 8 (8.4%) | 5 (17%) | 14 (27%) |  |
| Regression | |  |  | 0.505 |
| 1 | 2 (5.4%) | 0 (0%) | 1 (5.6%) |  |
| 2 | 8 (22%) | 1 (10%) | 3 (17%) |  |
| 3 | 8 (22%) | 6 (60%) | 6 (33%) |  |
| 4 | 19 (51%) | 3 (30%) | 8 (44%) |  |
| Unknown | 58 | 19 | 34 |  |
| Adjuvant Tx | 41 (46%) | 12 (44%) | 26 (50%) | 0.865 |
| Unknown | 6 | 2 | 0 |  |
| Recurrence | 30 (32%) | 9 (31%) | 20 (38%) | 0.667 |
| Site of Recurrence | |  |  | 0.148 |
| local | 5 (17%) | 1 (13%) | 2 (11%) |  |
| multiple | 9 (31%) | 1 (13%) | 9 (50%) |  |
| peritoneal | 0 (0%) | 0 (0%) | 2 (11%) |  |
| systemic | 15 (52%) | 6 (75%) | 5 (28%) |  |

| Supplementary Table 2: Intestinal Subtype | Adequate, N = 47*^1^* | Inadequate, N = 48*^1^* | p-value*^2^* |
| --- | --- | --- | --- |
| ASA |  |  | 0.18 |
| 1/2 | 22 (47%) | 16 (33%) |  |
| 3/4 | 25 (53%) | 32 (67%) |  |
| Age | 68.91 (10.75) | 68.69 (11.49) | 0.92 |
| Sex |  |  | 0.568 |
| male | 32 (68%) | 30 (63%) |  |
| female | 15 (32%) | 18 (38%) |  |
| Grade |  |  | 0.393 |
| well-moderate | 27 (66%) | 25 (57%) |  |
| poor | 14 (34%) | 19 (43%) |  |
| Unknown | 6 | 4 |  |
| cT-Stage | |  | 0.373 |
| cT1 | 3 (7.0%) | 8 (17%) |  |
| cT2 | 12 (28%) | 14 (30%) |  |
| cT3 | 22 (51%) | 21 (46%) |  |
| cT4 | 6 (14%) | 3 (6.5%) |  |
| Unknown | 4 | 2 |  |
| cN-Stage | |  | 0.464 |
| cN0 | 20 (43%) | 24 (51%) |  |
| cNpositive | 26 (57%) | 23 (49%) |  |
| Unknown | 1 | 1 |  |
| Neoadjuvant Treatment | 20 (43%) | 18 (38%) | 0.615 |
| Complication | 5 (11%) | 3 (6.3%) | 0.486 |
| Anastomotic Leakage | 2 (4.3%) | 2 (4.2%) | >0.999 |
| Lymph Nodes Resected | 29.32 (10.71) | 28.46 (15.80) | 0.148 |
| Proximal Margin Distance | 7.13 (2.24) | 2.74 (1.17) | <0.001 |
| pT-Stage | |  | 0.274 |
| pT0-1 | 15 (38%) | 16 (43%) |  |
| pT2 | 0 (0%) | 0 (0%) |  |
| pT3 | 18 (45%) | 19 (51%) |  |
| pT4 | 7 (18%) | 2 (5.4%) |  |
| Unknown | 7 | 11 |  |
| pN-Stage | |  | 0.436 |
| pN0 | 22 (47%) | 22 (46%) |  |
| pN1 | 6 (13%) | 7 (15%) |  |
| pN2 | 6 (13%) | 11 (23%) |  |
| pN3 | 13 (28%) | 8 (17%) |  |
| pM | 3 (6.4%) | 4 (8.3%) | >0.999 |
| Lymph Node Ratio | 0.17 (0.24) | 0.13 (0.19) | 0.885 |
| Resection Margin | |  | >0.999 |
| R0 | 43 (91%) | 44 (92%) |  |
| R1 | 4 (8.5%) | 4 (8.3%) |  |
| Regression | |  | 0.826 |
| 1 | 1 (5.0%) | 1 (5.9%) |  |
| 2 | 5 (25%) | 3 (18%) |  |
| 3 | 3 (15%) | 5 (29%) |  |
| 4 | 11 (55%) | 8 (47%) |  |
| Unknown | 27 | 31 |  |
| Adjuvant Tx | 24 (56%) | 17 (37%) | 0.075 |
| Unknown | 4 | 2 |  |
| Recurrence | 18 (38%) | 12 (25%) | 0.163 |
| Site of Recurrence | | | 0.112 |
| local | 5 (29%) | 0 (0%) |  |
| multiple | 4 (24%) | 5 (42%) |  |
| systemic | 8 (47%) | 7 (58%) |  |

| Supplementary Table 3: Mixed and Diffuse Types | Adequate, N = 231 | Inadequate, N = 581 | p-value2 |
| --- | --- | --- | --- |
| Signetgroup | |  | 0.31 |
| mixed | 6 (26%) | 23 (40%) |  |
| diffuse | 17 (74%) | 35 (60%) |  |
| ASA |  |  | 0.641 |
| 1/2 | 14 (61%) | 32 (55%) |  |
| 3/4 | 9 (39%) | 26 (45%) |  |
| Age | 61.39 (14.06) | 59.74 (12.32) | 0.479 |
| Sex (female) | 6 (26%) | 26 (45%) | 0.12 |
| Grade |  |  | 0.178 |
| well-moderate | 0 (0%) | 6 (11%) |  |
| poor | 22 (100%) | 51 (89%) |  |
| Unknown | 1 | 1 |  |
| cT-Stage | |  | 0.087 |
| cT1 | 5 (23%) | 4 (7.3%) |  |
| cT2 | 9 (41%) | 15 (27%) |  |
| cT3 | 7 (32%) | 29 (53%) |  |
| cT4 | 1 (4.5%) | 7 (13%) |  |
| Unknown | 1 | 3 |  |
| cN-Stage | |  | 0.025 |
| cN0 | 16 (73%) | 25 (45%) |  |
| cNpositive | 6 (27%) | 31 (55%) |  |
| Unknown | 1 | 2 |  |
| Neoadjuvant Treatment | 4 (17%) | 24 (41%) | 0.041 |
| Complication | 4 (17%) | 5 (8.6%) | 0.265 |
| Anastomotic Leakage | 1 (4.5%) | 1 (1.7%) | 0.477 |
| Lymph Nodes Resected | 24.83 (9.72) | 26.26 (11.80) | 0.645 |
| Proximal Margin Distance | 9.20 (1.46) | 3.98 (1.94) | <0.001 |
| pT-Stage | |  | 0.037 |
| pT0-1 | 10 (50%) | 9 (19%) |  |
| pT2 | 0 (0%) | 0 (0%) |  |
| pT3 | 7 (35%) | 28 (58%) |  |
| pT4 | 3 (15%) | 11 (23%) |  |
| Unknown | 3 | 10 |  |
| pN-Stage | |  | 0.067 |
| pN0 | 16 (70%) | 21 (36%) |  |
| pN1 | 3 (13%) | 12 (21%) |  |
| pN2 | 1 (4.3%) | 9 (16%) |  |
| pN3 | 3 (13%) | 16 (28%) |  |
| pM | 0 (0%) | 9 (16%) | 0.054 |
| Lymph Node Ratio | 0.07 (0.17) | 0.19 (0.25) | 0.007 |
| Resection Margin (R1) | 1 (4.3%) | 18 (31%) | 0.011 |
| Regression | |  | 0.294 |
| 1 | 0 (0%) | 1 (4.2%) |  |
| 2 | 2 (50%) | 2 (8.3%) |  |
| 3 | 1 (25%) | 11 (46%) |  |
| 4 | 1 (25%) | 10 (42%) |  |
| Unknown | 19 | 34 |  |
| Adjuvant Tx | 6 (26%) | 32 (57%) | 0.012 |
| Unknown | 0 | 2 |  |
| Recurrence | 2 (8.7%) | 27 (47%) | 0.001 |
| Site of Recurrence | | | 0.003 |
| local | 0 (0%) | 3 (13%) |  |
| multiple | 0 (0%) | 10 (42%) |  |
| peritoneal | 2 (100%) | 0 (0%) |  |
| systemic | 0 (0%) | 11 (46%) |  |

**Supplemental Text 1: Subgroup Analysis Node Negative**

In nodal negative and non-metastatic patients, 38 out of 80 (47.5%) had an adequate PMD. Except from complication rates (16% vs. 2.4%, p=0.049), there were no significant differences in clinicopathological characteristics between the cohorts with adequate versus inadequate PMD. Estimated 5-year survival was 79% and 81% for adequate and inadequate resection margin distance with no difference in OS (medians not reached, p=0.683) and TTP (medians not reached, p=0.251).

**Supplemental Text 2: Subgroup Analysis Node Positive**

Twenty-nine out of 70 (41.4%) nodal positive patients without distant metastatic disease had an adequate PMD. Significant differences in clinicopathological characteristics between the cohorts with adequate and inadequate PMD was only observed for histologic subtypes (intestinal 76% vs. 43%, p=0.016). Again, no significant differences were observed for median OS (adequate PMD: 94.8 months vs. inadequate PMD; 131.4 months, p=0.658) or median TTP (28.8 vs. 124.9 months, p=0.470).

**Supplemental Text 3: Subgroup Analysis R0**

In patients with negative resection margins, 65 out of 149 (43.6%) had an adequate PMD. No significant differences were found in clinicopathological characteristics. Estimated 5-year survival was 66% for patients with an adequate PMD and 69% with an inadequate PMD with no differences in OS (medians not reached, p=0.708) and TTP (medians not reached, p=0.541). The same effects as in the main analysis were also observed in patients with intestinal type cancer and R0 resections (mOS both not reached, p=0.514, mTTP both not reached, p=0.300, estimated 5-year survival adequate PMD 54% vs. inadequate PMD 69%). An association to improved disease specific survival was also observed in R0 resected patients with diffuse subtype (mTTP adequate PMD not reached vs. inadequate PMD 125 months, p=0.005), however, the difference in OS did not reach statistical significancy (mOS, not reached vs. 131.0 months, p=0.062 estimated 5-year survival 85% vs. 69%).
